# Shifting the paradigm in Evolve and Resequence studies: From analysis of single nucleotide polymorphisms to selected haplotype blocks

**DOI:** 10.1111/mec.14992

**Published:** 2019-02-22

**Authors:** Neda Barghi, Christian Schlötterer

**Affiliations:** ^1^ Institut für Populationsgenetik Vetmeduni Vienna Vienna Austria

**Keywords:** *drosophila*, experimental evolution, haplotype-block, linkage disequilibrium, pool-seq

## Abstract

For almost a decade the combination of whole genome sequencing with experimental evolution (Evolve and Resequence, E&R; Turner, Stewart, Fields, Rice, & Tarone, 2011) has been used to study adaptation in outcrossing organisms. However, complications caused by inversions and hitchhiking variants have prevented this powerful approach from living up to its potential. In this issue of *Molecular Ecology*, Michalak, Kang, Schou, Garner, and Loeschke (2018), provide an important step ahead by using a population of *Drosophila melanogaster* devoid of segregating inversions to identify the genetic basis of resistance to five environmental stressors. They further address the challenge of hitchhiking variants by reconstructing selected haplotype blocks. While it is apparent that the haplotype block reconstruction needs further refinements, their work underpins the potential of E&R studies in *Drosophila* to address fundamental questions in evolutionary biology.

Experimental evolution provides a powerful framework to study evolutionary processes in controlled environments while taking advantage of replicated populations under (almost) identical conditions. Furthermore, the potential to study the dynamics of evolutionary processes by the means of time series data makes experimental evolution particularly attractive. Recently, the combination of experimental evolution with whole genome sequencing of pooled individuals (Evolve and Resequence, E&R; Turner et al., 2011) has developed into a successful line of research studying the genetic architecture of adaptive traits (Schlötterer, Kofler, Versace, Tobler, & Franssen, 2015). *Drosophila melanogaster* is often used in E&R experiments because of its relatively short generation time and ease of maintenance in combination with sexual reproduction and access to natural populations (Schlötterer et al., 2015). While the phenotypic response in experimental *D. melanogaster* populations is usually fast and highly consistent across replicates (Burke et al., 2010), the large number of single nucleotide polymorphisms (SNPs) that appear to respond to selection makes interpretation of the genomic responses challenging (Burke et al., 2010; Franssen, Nolte, Tobler, & Schlötterer, 2015; Griffin, Hangartner, Fournier‐level, & Hoffmann, 2017; Turner et al., 2011). Many studies have focused on the analysis of these outlier SNPs, but it has become clear that there are far too many to be compatible with population genetic theory (Nuzhdin & Turner, 2014).

Pioneering work by Franssen et al., (2015) provided the first insights to explain this discrepancy. Their analysis of a thermally adapted *D. melanogaster* population showed that many of the candidate SNPs were located in genomic regions coinciding with inversions segregating in this population. Furthermore, they demonstrated that selection on low‐frequency haplotypes causes a strong selection signal not only for the target(s) of selection, but also for linked neutral SNPs, resulting in hitchhiking across several megabases. While recombination could break up the large selected haplotype blocks, the moderate number of recombination events in *Drosophila* experiments is not enough for this to occur. Thus, the combination of segregating inversions with selection on low‐frequency haplotypes could explain the large number of candidate SNPs in *D. melanogaster* E&R studies (Franssen et al., 2015; Nuzhdin & Turner, 2014).

Another potential confounding factor contributing to the excessive number of candidate SNPs in E&R studies, which has not yet been studied in detail, is the widespread use of laboratory‐adapted founder populations. Such populations have been maintained at rather large census population sizes for many years (e.g. Burke et al., 2010; Turner et al., 2011) to facilitate adaptation to laboratory conditions. While this procedure circumvents the problem of confounding adaptation to laboratory conditions with the adaptive response to the selection treatment, it creates the potential problem of reduced haplotype diversity in the founder population (Figure 1).

**Figure 1:**
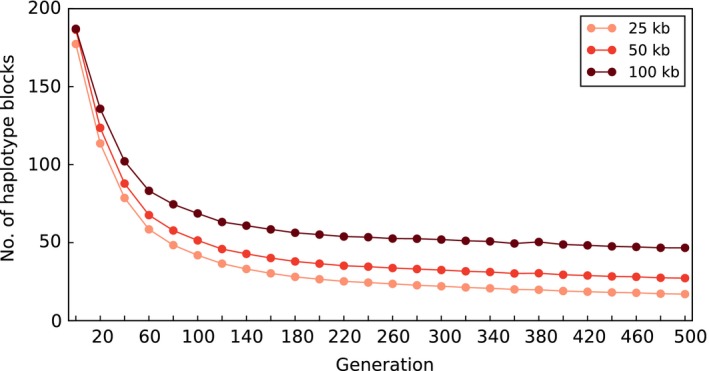
Reduction of haplotype diversity in populations maintained for many generations without selection. We simulated 1,037,324 SNPs on chromosome 2L in a population of 1,000 diploid individuals for 500 generations using 189 founder haplotypes Howie et al., (2018) and *D. melanogaster* recombination rate (Comeron et al., 2012). Computer simulations were performed using MimicrEE2 (Vlachos & Kofler, 2018). The number of haplotypes in 25‐, 50‐ and 100‐kb regions are shown. The reported haplotype diversity is conservative because haplotype blocks differing by only a single SNP are treated as distinct

Michalak et al., (2018) studied the adaptive response of a freshly collected *D. melanogaster* population to five different selection treatments (heat shock, heat knockdown, starvation, cold shock and desiccation). Unlike in previous studies, the founder population used by Michalak et al., (2018) was almost free of segregating inversions. Consequently, they observed clear selection signatures: several distinct peak structures emerged on Manhattan plots based on Cochran–Mantel–Haenszel (CMH) tests across five replicate populations (figure 5 in Michalak et al., 2018).

Given the apparent problems caused by segregating inversions in *D. melanogaster*, we recently proposed to establish *Drosophila simulans* as an alternative model for experimental evolution (Barghi, Tobler, Nolte, & Schlötterer, 2017). This species lacks segregating inversions and has a higher recombination rate, which remains almost uniform across entire chromosome arms (Howie, Mazzucco, Taus, & Schlötterer, 2018), providing a higher resolution in E&R studies. The advantage of *D. simulans* was confirmed in a recent E&R experiment studying temperature adaptation in the species, which resulted in identification of a few distinct selection signatures (Mallard, Nolte, Tobler, Kapun, & Schlötterer, 2018). The results of Michalak et al., (2018) show that future high‐resolution E&R studies are not restricted to *D. simulans*, for which functional downstream analyses are much more difficult, but that freshly collected *D. melanogaster *populations are a viable alternative, but only if they lack segregating inversions.

Michalak et al., (2018) also make an important step ahead to account for the other challenge of E&R studies, linkage disequilibrium (LD) between neutral SNPs and selection target(s), which inflates the number of candidate SNPs due to hitchhiking. Following an approach pioneered by (Franssen, Barton, & Schlötterer, 2017), they reconstructed selected haplotype blocks based on the correlation of allele frequencies of linked SNPs across replicates and time points. Michalak et al., (2018) obtained 314 selected haplotypes across five selection regimes. A closer inspection shows that a substantial number of their outlier SNPs fall into genomic regions overlapping with a single selected haplotype block (figure 5 in Michalak et al., 2018). This confirms that haplotype‐based analyses are more informative—rather than hundreds or thousands of putative selected targets, the selection response can be explained by tens to hundreds of adaptive alleles residing on selected haplotypes, as predicted before (Nuzhdin & Turner, 2014). Similar problems have been identified in experimental evolution studies using other species such as yeast and *Caenorhabditis elegans*.

Nevertheless, the haplotype‐based analysis of Michalak et al., (2018) requires further improvements; many different haplotype blocks are identified next to each other (figure 5 in Michalak et al., 2018). This problem was also noted by Barghi et al., 2019, who showed that selection targets with higher starting frequencies typically occur on multiple haplotypes. When too stringent clustering is applied (i.e. high correlation), multiple haplotype blocks are identified despite being affected by a single target of selection. Barghi et al., (2019) addressed this by a two‐step clustering procedure and confirmed their clustering with experimentally phased haplotypes from evolved populations. We illustrate the possible nonindependence of adjacent haplotype blocks identified in Michalak et al., (2018) by plotting their frequency trajectories in two selection regimes (Figure 2). This analysis shows that SNPs in these haplotype blocks have highly correlated allele frequency trajectories, suggesting that the number of selected targets is potentially considerably lower than implied by the clustering analysis of Michalak et al., (2018). Hence, it is clear that a robust inference of selection targets cannot be restricted to the identification of outlier SNPs or peaks in a Manhattan plot. Rather, a shift from the analysis of individual SNPs to the analysis of selected haplotype blocks is inevitable.

**Figure 2:**
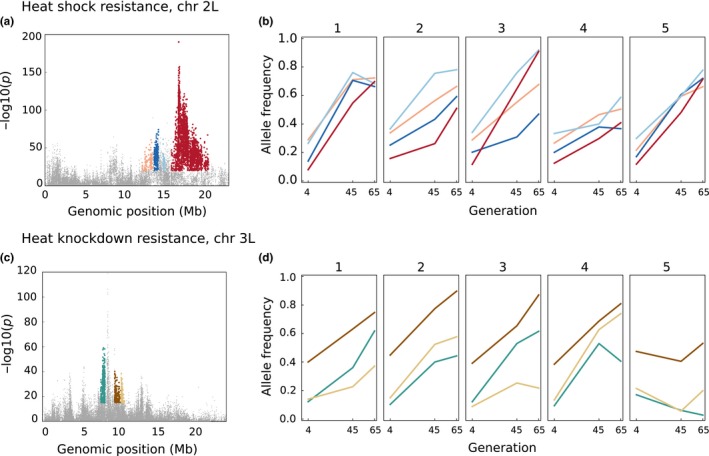
Nonindependence of selected haplotype blocks reconstructed by (Michalak et al., 2018). (a,c) Manhattan plots of the negative log_10_‐transformed *p*‐values from CMH tests contrasting five replicate populations at F4 with F65 for (a) heat shock resistance selection (chromosome arm 2L) and (c) heat knockdown resistance selection (chromosome arm 3L). SNPs in reconstructed haplotype blocks (a: blocks 9–12, c: blocks 25, 30 and 32) are shown in block‐specific colours. (b,d) Median allele frequency trajectories of SNPs with CMH negative log_10_‐transformed *p*‐value ≥20 (a) or ≥15 (c) in haplotype blocks in panels (a) and (c) (colour code corresponds to panels (a) and (c), respectively) in replicates 1–5. Despite different starting frequencies, the median trajectories of adjacent blocks resemble each other, suggesting linkage disequilibrium and possibly joint selection target(s)

Unfortunately, it is not yet clear which haplotype reconstruction method is the best. First, when the founder haplotypes are known, evolved haplotypes can be reconstructed computationally (Kessner, Turner, & Novembre, 2013). However, as only a moderate number of sequenced founder lines are available (Lack, Lang, Tang, Corbett‐Detig, & Pool, 2016; Mackay et al., 2012) the choice of founder populations is very limited. Second, statistical phasing of heterozygous individuals from evolved generations allows the identification of haplotype blocks containing selected target(s). Currently, the power of this approach in obtaining reliable haplotypes is not clear; a recent analysis indicated that the switch error rates in natural *D. melanogaster* populations are prohibitively high (Bukowicki, Franssen, & Schlötterer, 2016). Third, evolved haplotypes can be phased experimentally by sequencing single F_1_ individuals from crosses between the target strains and an inbred reference (Barghi et al., 2019; Franssen et al., 2015). Although highly accurate, this method requires live material for crosses. Finally, improving the correlation analysis of Franssen et al., (2017) could potentially increase the accuracy of identified target(s) of selection.

Regardless of the exact methods being used in future analyses of E&R studies, the study of Michalak et al., (2018) provides firm evidence that E&R using *Drosophila* bears a huge potential to provide unprecedented insights into the genetic architecture of adaptation.
